# Optimized multi-echo gradient-echo magnetic resonance imaging for gray and white matter segmentation in the lumbosacral cord at 3 T

**DOI:** 10.1038/s41598-022-20395-1

**Published:** 2022-10-03

**Authors:** Silvan Büeler, Marios C. Yiannakas, Zdravko Damjanovski, Patrick Freund, Martina D. Liechti, Gergely David

**Affiliations:** 1 Department of Neuro-Urology, Balgrist University Hospital, University of Zurich, Forchstrasse 340, 8008 Zurich, Switzerland; 2 NMR Research Unit, Queen Square MS Centre, Department of Neuroinflammation, UCL Queen Square Institute of Neurology, University College London, London, UK; 3 Spinal Cord Injury Center, Balgrist University Hospital, University of Zurich, Forchstrasse 340, 8008 Zurich, Switzerland; 4 Department of Neurophysics, Max Planck Institute for Human Cognitive and Brain Sciences, Leipzig, Germany; 5 Wellcome Trust Centre for Neuroimaging, UCL Queen Square Institute of Neurology, London, UK

**Keywords:** Neurodegeneration, Spinal cord diseases, Amyotrophic lateral sclerosis, Spine structure, Amyotrophic lateral sclerosis, Multiple sclerosis, Neurodegeneration

## Abstract

Atrophy in the spinal cord (SC), gray (GM) and white matter (WM) is typically measured in-vivo by image segmentation on multi-echo gradient-echo magnetic resonance images. The aim of this study was to establish an acquisition and analysis protocol for optimal SC and GM segmentation in the lumbosacral cord at 3 T. Ten healthy volunteers underwent imaging of the lumbosacral cord using a 3D spoiled multi-echo gradient-echo sequence (Siemens FLASH, with 5 echoes and 8 repetitions) on a Siemens Prisma 3 T scanner. Optimal numbers of successive echoes and signal averages were investigated comparing signal-to-noise (SNR) and contrast-to-noise ratio (CNR) values as well as qualitative ratings for segmentability by experts. The combination of 5 successive echoes yielded the highest CNR between WM and cerebrospinal fluid and the highest rating for SC segmentability. The combination of 3 and 4 successive echoes yielded the highest CNR between GM and WM and the highest rating for GM segmentability in the lumbosacral enlargement and conus medullaris, respectively. For segmenting the SC and GM in the same image, we suggest combining 3 successive echoes. For SC or GM segmentation only, we recommend combining 5 or 3 successive echoes, respectively. Six signal averages yielded good contrast for reliable SC and GM segmentation in all subjects. Clinical applications could benefit from these recommendations as they allow for accurate SC and GM segmentation in the lumbosacral cord.

## Introduction

Spinal cord injury (SCI) and many neurological disorders such as multiple sclerosis, amyotrophic lateral sclerosis, or multiple system atrophy can severely affect the lumbosacral cord, often leading to the loss of lower limb function and sensation, as well as impairment of bladder, bowel, and sexual function^1–5^. The culmination of pathophysiological processes such as demyelination and axonal degeneration has been shown to result in irreversible tissue loss (i.e., atrophy) in traumatic SCI^6^, multiple sclerosis^7^ and amyotrophic lateral sclerosis^8^. Atrophy of the lumbosacral cord can be quantified indirectly in vivo by measuring the cross-sectional area of the spinal cord (SC), gray matter (GM), or white matter (WM) from images acquired in the axial plane with the use of magnetic resonance imaging (MRI)^9–16^.

Multi-echo gradient-echo sequences such as FLASH, MEDIC (Siemens), mFFE (Philips), MERGE (GE), to name a few, offer a mixture of T1, proton density (PD), and T2*-weighting and provide good contrast between GM, WM, and cerebrospinal fluid (CSF), thus facilitating volumetric measurements of tissue compartments^17–20^. For the cervical cord, these sequences have been in use in clinical studies to produce high-resolution axial images suitable for segmentation and cross-sectional area measurements^21–24^. For the lumbosacral cord, the feasibility of SC and GM segmentation has been demonstrated with low intra- and inter-rater variability in the lumbosacral enlargement (LSE)^25^ and the conus medullaris (CM)^26^. First clinical applications in this region revealed evidence of remote atrophy in the LSE after cervical SCI^27–29^ and degenerative cervical myelopathy^30^.

Segmentation of SC and GM relies on high contrast-to-noise ratio (CNR) between WM and CSF and between GM and WM, which in turn depends on the choice of sequence parameters. Among others, echo times, number of echoes, and number of signal averages (NSA) have profound effects on the CNR and the signal-to-noise ratio (SNR), and therefore on the accuracy and precision of the segmentation. While early echoes within a multi-echo acquisition are predominantly T1- and PD-weighted with strong GM/WM contrast, later echoes add more T2*-weighting, enhancing WM/CSF and lowering GM/WM contrast, but also introducing more off-resonance artifacts due to longer read-out times^17^. Furthermore, CNR and SNR scale with the square root of NSA^31^. Therefore, practical recommendations are necessary for routine applications by compromising between GM/WM and WM/CSF contrast and trading off SNR against imaging time. However, unlike in the cervical cord^32^, there is currently no clear consensus on the sequence parameters to be used in the lumbosacral cord, limiting the accuracy and reproducibility of volumetric assessments, the comparability of studies, and hindering the clinical adoption of these sequences.

In this study, the feasibility of SC and GM segmentation was investigated in the healthy lower spinal cord using a 3 T MRI system and a 3D multi-echo gradient-echo sequence, by accounting for key technical variables (number of echoes and NSA) pertinent to most product sequence variations utilized within clinical setting. Building on both quantitative (i.e., SNR, CNR calculations) and qualitative (i.e., independent ratings) analyses, we aimed to provide recommendations for future clinical investigations.

## Materials and methods

### Study participants

Ten healthy participants (mean (± SD) age: 33.1 ± 11.9 years, range: 22–55, 6 female) were recruited for this study. Inclusion criteria were (i) no MRI contraindications, (ii) no history of neurological and mental disorders, and (iii) > 18 years of age. Written informed consent was obtained from all study participants. The study was approved by the local ethics committee (Kantonale Ethikkommission Zürich, BASEC-Nr. 2019-00074) and conducted in accordance with the Declaration of Helsinki.

### MRI acquisition

**Figure 1:**
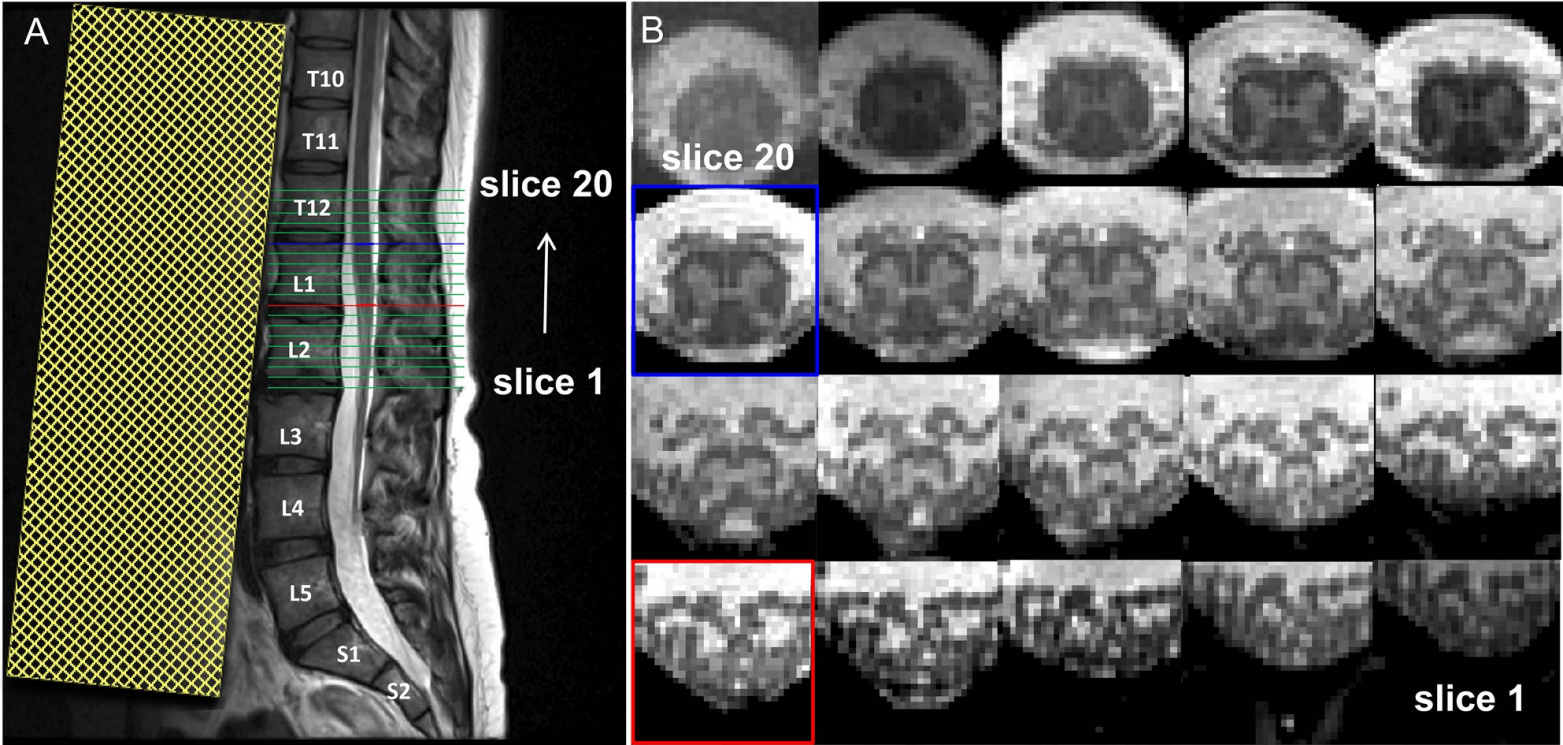
(**A**) Sagittal T2-weighted turbo spin echo acquisition in the lower spine used for subsequent prescription of the high-resolution axial acquisition. (**B**) Corresponding axial slices acquired with the 3D multi-echo gradient-echo sequence ( Siemens FLASH) in the caudal-rostral direction (slices 1–20). Highlighted are the slice in the lumbosacral enlargement (LSE) with the largest cord cross-sectional spinal cord area (defined as the "LSE slice" and shown in light blue in **A** and **B**; here: slice 15), and the most caudal slice in the conus medullaris (CM) where the gray matter still has the characteristic butterfly shape (defined as the "CM slice" and shown in red in **A** and **B**; here: slice 9). A saturation band, displayed as yellow shaded area in A, was placed anterior to the spine to suppress signal and possible artifacts arising from abdominal peristalsis.

Scanning was performed on a 3 T Siemens Prisma MRI scanner (Siemens Healthineers, Erlangen, Germany) equipped with a body transmit coil and a 32-channel spine matrix coil. Motion in the lower back area was reduced by (i) placing a spine vacuum cushion below the legs, (ii) applying Velcro straps around the knees, hips, and chest, and (iii) instructing the participants to breath from the chest rather than from the abdomen. In addition, foam wedges were placed below the knees to minimize the lower spine natural lordotic curve and to maximize the contact between the lower back and spine matrix coil. A sagittal T2-weighted turbo spin echo (TSE) sequence with 15 slices of 4 mm thickness (10% slice gap) was acquired as an anatomical reference of the lumbosacral cord, encompassing the LSE and CM, to facilitate slice prescription of the main high-resolution acquisition in the axial plane (Fig. 1A). Additional sequence parameters for the sagittal T2-weighted TSE included an in-plane resolution of 0.7 × 0.7 mm^2^, field of view of 330 × 330 mm^2^, repetition time of 3 s, echo time of 89 ms, flip angle of 154°, and acquisition time of 00:59 min. Then, a 3D spoiled multi-echo gradient-echo sequence (Siemens FLASH) with 20 axial-oblique slices of 5 mm thickness (no gap) was acquired with the slice stack positioned perpendicularly to the longitudinal axis of the cord, ensuring coverage of both the LSE and CM in all cases (Fig. 1B). Additional sequence parameters for the 3D FLASH included an in-plane resolution of 0.5 × 0.5 mm^2^, field of view of 192 × 192 mm^2^, repetition time of 38 ms, echo train length of 5, first echo time of 6.85 ms, echo spacing of 4 ms, flip angle of 8°, 8 individual repetitions (i.e., without k-space averaging), GRAPPA 2x, no partial Fourier, no navigator echoes, bandwidth of 260 Hz/pixel, and acquisition time of 17:56 min.

### Image processing and segmentation

**Figure 2:**
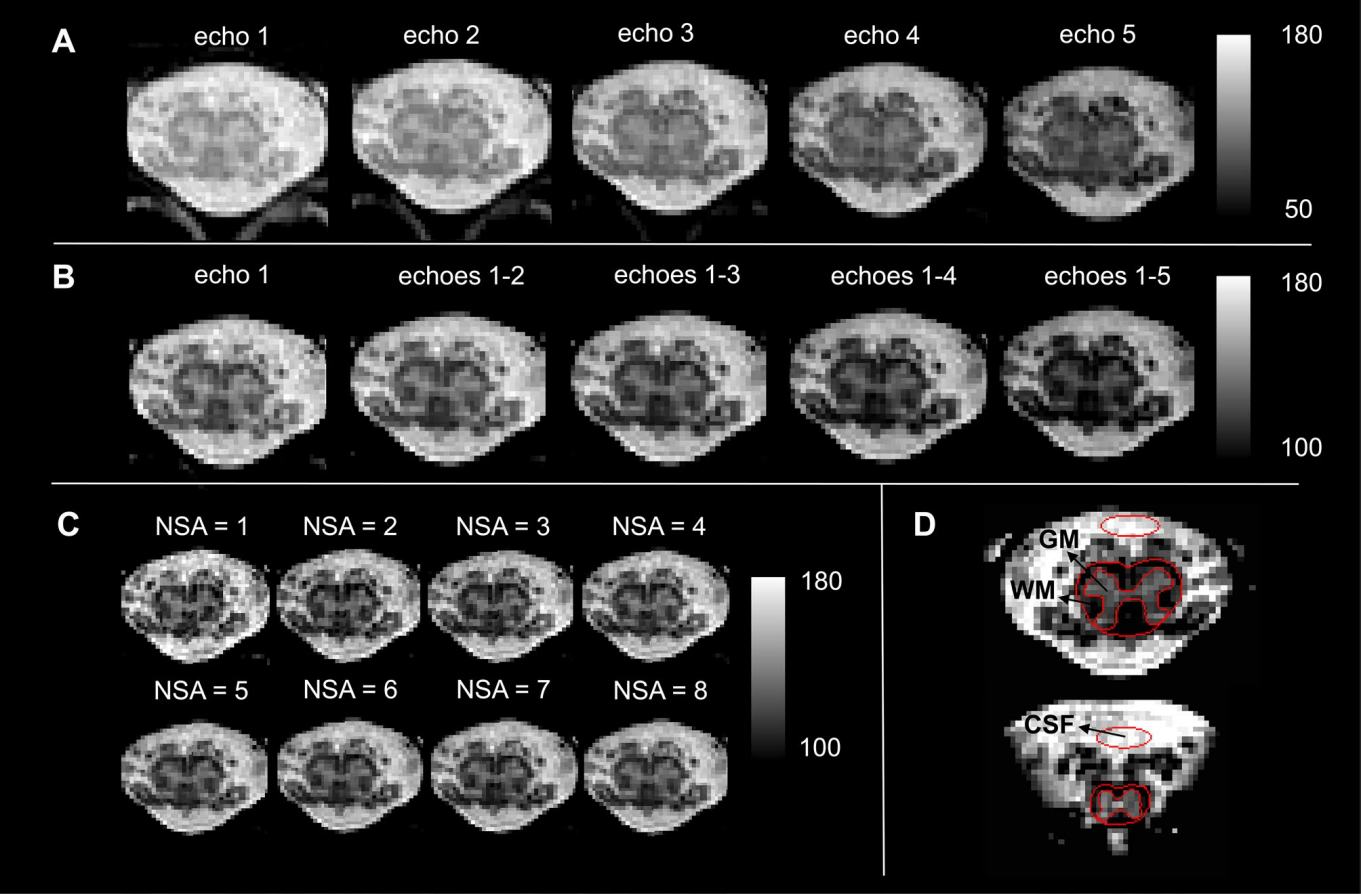
Visual representation of echoes, echo combinations, signal averages, and image segmentations. The 3D spoiled multi-echo gradient echo sequence (Siemens FLASH) consisted of 5 echoes and was acquired with 8 individual repetitions. For each subject, a series of images was created by successively combining echoes (echo 1, 1–2, 1–3, 1–4, 1–5) and averaging across repetitions (number of signal averages (NSA): 1, 2, 3, …, 8), resulting in a total of 72 images. (**A**) Image series of individual echoes (NSA = 8) for a representative slice in the lumbosacral enlargement (LSE). (**B**) Image series of increasing number of combined echoes in the same slice as in (**A**) (NSA = 8). (**C**) Image series with increasing NSA (3 combined echoes). (**D**) Spinal cord (SC) and gray matter (GM) were segmented manually in each slice (here a representative LSE and conus medullaris slice are shown). A mask of cerebrospinal fluid (CSF) was drawn anterior to the SC. White matter (WM) mask was obtained by subtracting GM from the SC mask.

In each participant, the 3D FLASH sequence yielded 40 images corresponding to 5 echoes in each of the 8 individual repetitions. A series of new images were created by (i) combining increasing number of successive echoes via root-mean-squares, yielding 4 more images for each repetition with 2, 3, 4, and 5 combined echoes, and (ii) averaging increasing number of successive repetitions yielding 7 more images for each echo and echo combination with NSA ranging from 2 to 8 (Fig. 2A–C). Motion among individual repetitions was assessed by the ECMOCO motion correction algorithm^33^ (part of the SPM12-based ACID toolbox) with three degrees of freedom (translation only). The absolute displacement of each repetition was defined as the square root of the sum of squared translations along x, y, and z directions between the actual repetition and the mean of all repetitions. We used the maximum absolute displacement (i.e., the maximum of the absolute displacements among all repetitions) to quantify the maximum extent of motion within a dataset. Note that motion correction was run only for quality assessment purposes; co-registration between individual repetitions was not required as absolute displacements were negligible in all subjects.

SC and GM were segmented in a single reference image (3 combined echoes, NSA = 8) using the manual sub-voxel segmentation tools in JIM 7.0 (Xinapse systems, http://www.xinapse.com). A CSF mask was created manually by placing an ellipsoidal region of interest (ROI) anterior to the SC with an area of 7.5 mm^2^, which ensured no inclusion of nerve roots in all participants (Fig. 2D). Sub-voxel segmentations were binarized at an inclusion threshold of 100% for SC and 50% for GM and CSF. A binary WM mask was created by subtracting the binary GM mask from the binary SC mask.

### Quantitative comparison

For image comparisons, we computed (i) SNR within the binary WM and GM masks, (ii) CNR between GM/WM and WM/CSF, and (iii) contrast between GM/WM and WM/CSF. SNR within a ROI was computed as the mean intensity ($I$) within the ROI relative to its standard deviation: $SNR=\frac{mea{n}_{ROI}(I)}{{SD}_{ROI}(I)}$, while CNR was computed as the absolute difference in mean $I$ between ROI_1_ and ROI_2_, divided by the square root of the sum of their standard deviation: $CNR=\frac{|mean\left({\mathrm{I}}_{ROI,1}\right)-mean\left({I}_{ROI,2}\right)|}{\sqrt{SD{\left({\mathrm{I}}_{ROI,1}\right)}^{2}+SD{\left({\mathrm{I}}_{ROI,2}\right)}^{2}}}$. Contrast between ROI_1_ and ROI_2_ was defined as $\frac{|mean\left({\mathrm{I}}_{ROI,1}\right)-mean\left({I}_{ROI,2}\right)|}{\frac{1}{2} \left(mean\left({\mathrm{I}}_{ROI,1}\right) + mean\left({I}_{ROI,2}\right)\right)}$. Values of ${SNR}_{WM}$, ${SNR}_{GM}$, $CN{R}_{WM/CSF}$, $CN{R}_{GM/WM}$, $CN{R}_{WM/CSF}/\sqrt{t}$ (with t representing the acquisition time), $CN{R}_{GM/WM}/\sqrt{t}$, GM/WM and WM/CSF contrast were computed slice-wise and were averaged across two distinct slice stacks: (i) in three slices around the "LSE slice" defined as the slice with the greatest cross-sectional SC area as measured by manual segmentation and (ii) in three slices around the "CM slice" defined as the most caudal slice where the GM still has the characteristic butterfly shape. Images with higher values of CNR and SNR were considered to indicate better segmentability. As all echo combinations are expected to follow a square-root dependency on NSA, the influence of NSA on quantitative imaging metrics was analyzed only for 3 combined echoes, which were considered to be the most representative images for the dataset.

### Qualitative comparison

Successful segmentation depends not only on signal and contrast properties but also on other factors such as the visualization of a clear border at the tissue interface and the absence of image artifacts. Therefore, in each participant, the 5 echoes and 4 echo combinations (with NSA = 8 each) were scored in terms of segmentability by 5 experienced raters (S.B., M.C.Y., Z.D., M.D.L., G.D.). The raters assigned a ranking score to each image ranging from 1 (worst segmentable image) to 9 (best segmentable image). Images were scored separately per type of segmentation including SC only segmentability, GM only segmentability, and joint SC and GM segmentability. For each participant, scoring was performed in two regions, the "LSE slice" and the "CM slice" defined in section “Quantitative comparison”. In total, each rater submitted six sets of scores for each participant (three types of segmentation and two regions). The assigned ranking scores were averaged across raters and participants to obtain a mean ranking score per echo/echo combination, type of segmentation, and region.

### Statistical analysis

Interval scaled metrics were summarized with means and standard deviations (SD). Statistical analysis was performed for the metrics that are most closely associated with image segmentability ($CN{R}_{WM/CSF}$, $CN{R}_{GM/WM}$, and ranking scores for segmentability); for other metrics, we applied descriptive statistics. To analyze the effect of number of combined echoes (1, 1–2, 1–3, 1–4, 1–5), region (LSE, CM), and their interaction on $CN{R}_{WM/CSF}$ and $CN{R}_{GM/WM}$, a two-way repeated measures ANOVA was performed with the number of combined echoes and the region as within-subject variables. To analyze the effect of number of combined echoes on the ranking score of SC, GM and joint SC and GM segmentability, a three-way repeated measures ANOVA was performed with the rater as additional within-subject variable. Pairwise differences between echo combinations were tested using Tukey's post-hoc test for multiple comparison (p < 0.05).

## Results

Quality assessment revealed negligible motion artifacts, with maximum absolute displacement between individual repetitions below 0.6 mm (little larger than a single voxel) in all subjects. SC and GM were visible and segmentable in all subjects. A summary of $CN{R}_{WM/CSF}$, $CN{R}_{GM/WM}$, GM/WM contrast, WM/CSF contrast, ${SNR}_{GM}$, ${SNR}_{WM}$, $CN{R}_{WM/CSF}/\sqrt{t}$, and $CN{R}_{GM/WM}/\sqrt{t}$ values are shown for individual and combined echoes in Supplementary Table S1 and for different values of NSA in Supplementary Table S2 (online).

### The influence of echoes and echo combinations on quantitative imaging metrics

**Figure 3:**
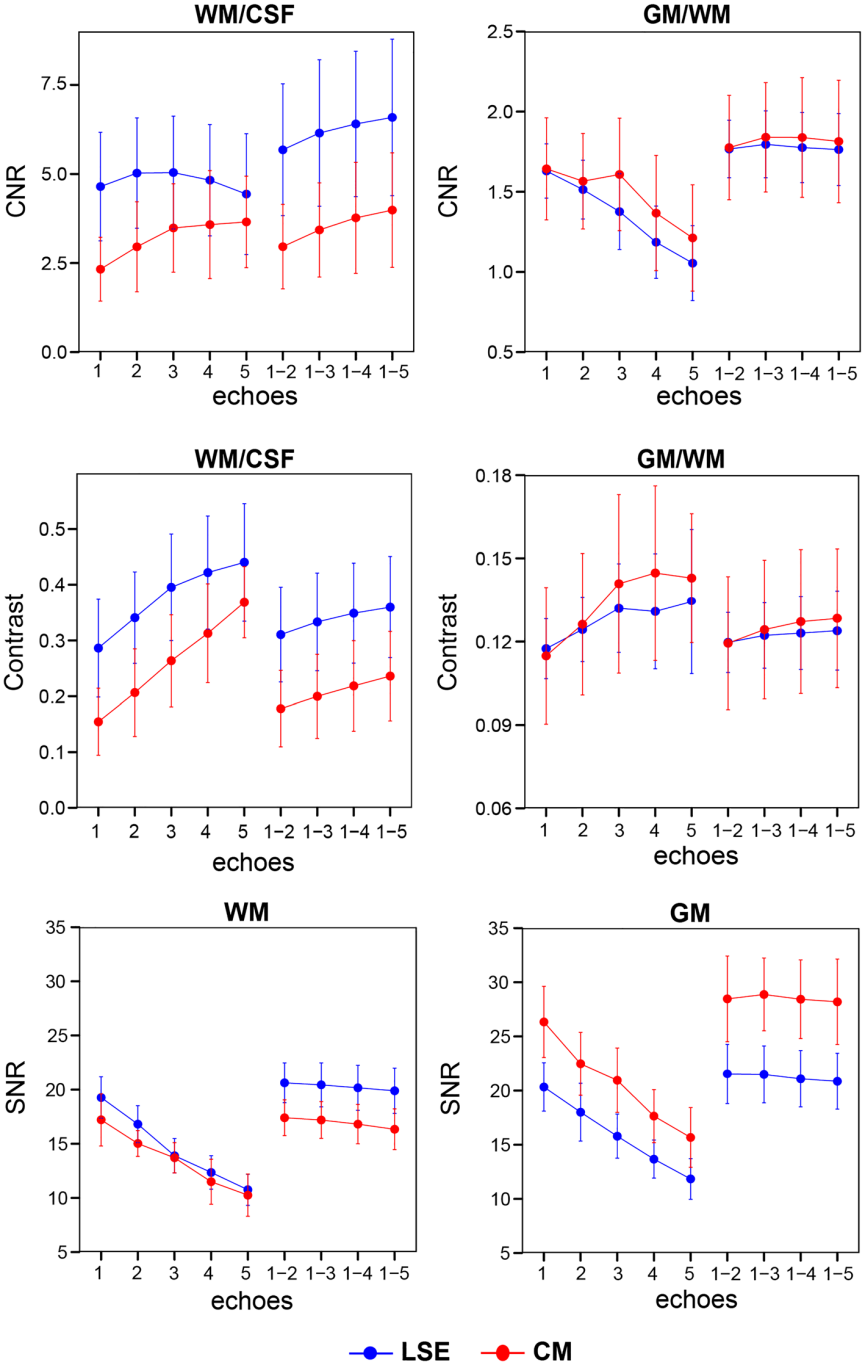
Quantitative comparison of the individual and combined echoes (8 signal averages) in terms of contrast-to-noise ratio (CNR) and contrast between white matter (WM) and cerebrospinal fluid (CSF) and betwen gray matter (GM) and WM, and signal-to-noise ratio (SNR) of WM and GM. Values are displayed for individual echoes (1, 2, 3, 4, and 5) and combined echoes (1–2, 1–3, 1–4 and 1–5). All measures are displayed separately for the lumbosacral enlargement (LSE) in blue and conus medullaris (CM) in red. In all subplots, error bars represent standard deviation across participants (n = 10).

For each echo and echo combination with NSA of 8, quantitative metrics including $CN{R}_{WM/CSF}$, $CN{R}_{GM/WM}$, GM/WM contrast, WM/CSF contrast, ${SNR}_{WM}$, and ${SNR}_{GM}$ are displayed in Fig. 3. On $CN{R}_{WM/CSF}$, ANOVA revealed a significant effect of the number of combined echoes (F(4,36) = 58.98, p < 0.001) and the region (F(1,9) = 15.87, p = 0.003), but no significant interaction between them (albeit higher values in the LSE). The highest $CN{R}_{WM/CSF}$ value was found when combining 5 successive echoes (all acquired echoes) (LSE: 6.59 ± 2.20, CM: 3.99 ± 1.61), which showed significant pairwise differences to the first echo (LSE & CM: p < 0.001), 2 combined echoes (LSE & CM: p < 0.001), and in the CM also to 3 combined echoes (p = 0.042).

$CN{R}_{GM/WM}$, ANOVA revealed a significant effect of the number of combined echoes (F(4,36) = 19.92, p < 0.001) but not of the region and their interaction (Fig. 3 upper row). All echo combinations had higher $CN{R}_{GM/WM}$ values than the first echo alone (p < 0.01). The highest value was found when combining 3 and 4 successive echoes in the LSE (1.80 ± 0.21) and CM (1.84 ± 0.37), respectively, although they were not significantly different to other echo combinations.

WM/CSF contrast showed a steady and sharp increase with later echoes, with higher values in the LSE than in the CM (Fig. 3 middle row). Among all echoes and echo combinations, the fifth echo had the highest value for both LSE (0.440 ± 0.105) and CM (0.369 ± 0.064. GM/WM contrast increased only moderately with later echoes, with similar values in both regions. Among all echoes and echo combinations, the fifth echo had the highest value for both LSE (0.134 ± 0.026) and CM (0.143 ± 0.023).

${SNR}_{WM}$ decreased with later echoes and had slightly higher values in the LSE than in the CM (Fig. 3, bottom row). Echo combinations had higher values than individual echoes, with 2 combined echoes having the highest value for both LSE (20.6 ± 1.8) and CM (17.4 ± 1.6). ${SNR}_{GM}$ also decreased with later echoes but, unlike ${SNR}_{WM}$, showed higher values in the CM compared to the LSE. Again, higher values were reported for combined echoes, with 3 combined echoes having the highest value for both LSE (21.5 ± 2.6) and CM (28.9 ± 3.4).

### The influence of number of signal averages (NSA) on quantitative imaging metrics

**Figure 4:**
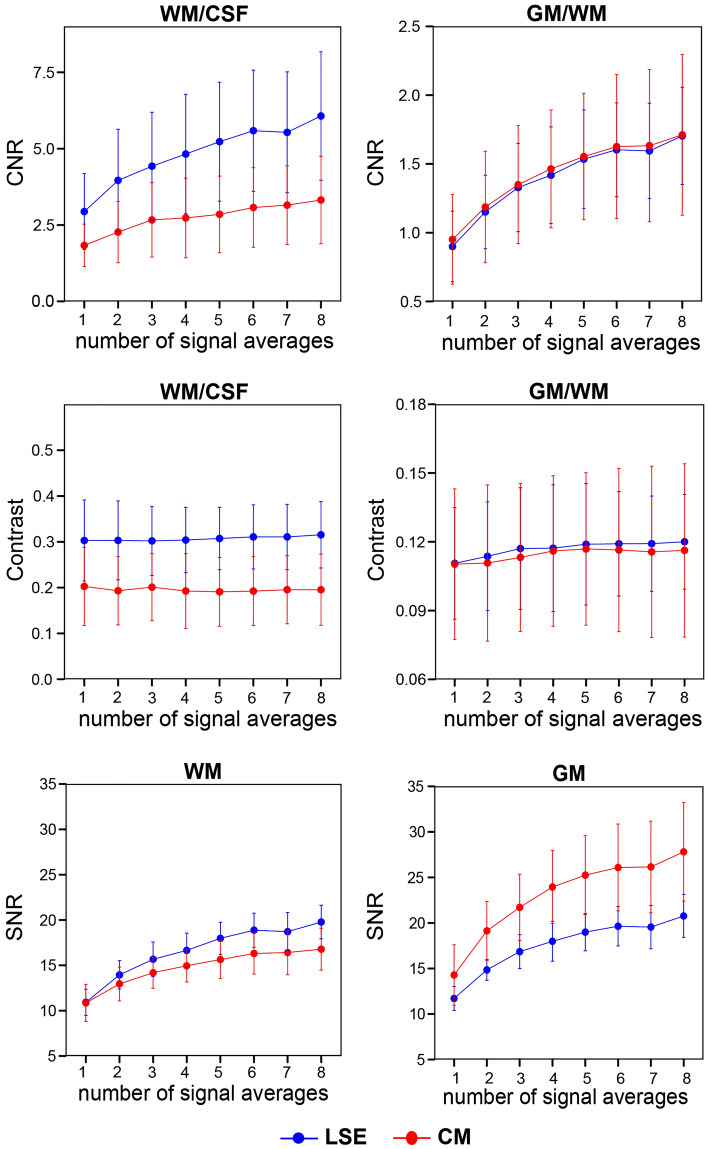
Quantitative comparison of the number of signal averages (NSA) (3 combined echoes) in terms of contrast-to-noise ratio (CNR) and contrast between white matter (WM) and cerebrospinal fluid (CSF) and between gray matter (GM) and WM, and signal-to-noise ratio (SNR) of WM and GM. All measures are displayed separately for the lumbosacral enlargement (LSE) in blue and conus medullaris (CM) in red. In all subplots, error bars represent standard deviation across participants (n = 10).

For each NSA with 3 combined echoes, quantitative metrics including $CN{R}_{WM/CSF}$, $CN{R}_{GM/WM}$, WM/CSF contrast, GM/WM contrast, ${SNR}_{WM}$, and ${SNR}_{GM}$ are displayed in Fig. 4. With increasing NSA, $CN{R}_{WM/CSF}$, $CN{R}_{GM/WM}$, ${SNR}_{WM}$ and ${SNR}_{GM}$ followed an approximate square-root dependency with incrementally smaller improvements. Accordingly, NSA of 8 had the highest $CN{R}_{WM/CSF}$ (6.07 ± 2.10 for LSE, 3.32 ± 1.43 for CM) and $CN{R}_{GM/WM}$ (1.70 ± 0.35 for LSE, 1.71 ± 0.58 for CM). WM/CSF and GM/WM contrast stayed largely constant for all NSA, with WM/CSF contrast around 0.3 in the LSE and 0.2 in the CM, and GM/WM contrast around 0.15 and 0.10 in the LSE and CM, respectively.

### Qualitative analyses

**Figure 5:**
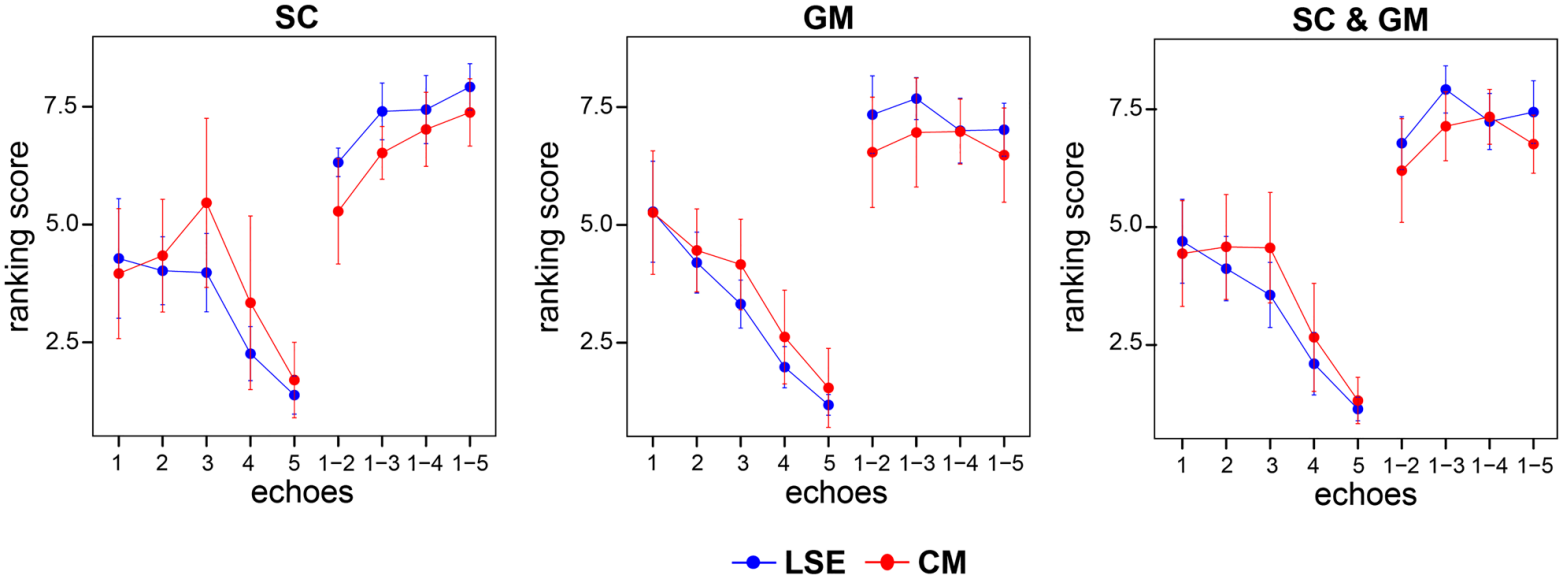
Qualitative comparison of the individual and combined echoes (8 signal averages). Shown are ranking scores assigned to individual echoes (1, 2, 3, 4, 5) and combined echoes (1–2, 1–3, 1–4, 1–5) and averaged across all participants (n = 10) and raters (n = 5). Echoes/echo combinations were ranked separately for spinal cord (SC) only, gray matter (GM) only and joint SC and GM segmentability. The analysis was performed in a representative slice at the lumbosacral enlargement (LSE) in blue and conus medullaris (CM) in red. Ranking score ranges from 1 (lowest score) to 9 (highest score). Error bars represent standard deviation across participants.

According to the raters’ rankings, the segmentability of the LSE was more difficult in the individual echoes, particularly in the later echoes, which can be seen in Fig. 5 as a steady decrease in the mean ranking score from echo one to five for each type of segmentation (SC, GM, and joint SC and GM segmentability). The number of combined echoes had a significant effect on the ranking score for SC (F(4,36) = 56.94, p < 0.001), GM (F(4,36) = 14.63, p < 0.001), and joint SC and GM segmentability (F(4,36) = 51.33, p < 0.001). The highest ranking score for SC segmentation was found when combining 5 successive echoes, which showed significant pairwise differences to the first echo (LSE & CM: p < 0.001) and 2 combined echoes (LSE & CM: p < 0.001). The highest ranking scores for GM and joint SC and GM segmentability were obtained when combining 3 and 4 successive echoes in the LSE and CM, respectively, which were significantly different to the first echo (GM and joint SC and GM: p < 0.001) and 2 combined echoes (joint SC and GM: p = 0.013).

## Discussion

In this study, we aimed to establish an optimized multi-echo gradient-echo data acquisition scheme for tissue-specific assessment of the lumbosacral cord at 3 T. Individual echoes in a multi-echo gradient-echo sequence have different signal, contrast, and noise properties. While the contributions of PD and T1 contrast remain the same across echoes, the contribution of T2* (and T2) contrast increases in later echoes, making early echoes predominantly PD and T1-weighted and later echoes increasingly T2*-weighted. We found the combination of 5 successive echoes (all available echoes) to be optimal for SC segmentation and the combination of 3 (lumbosacral enlargement) or 4 successive echoes (conus medullaris) to be optimal for GM segmentation.

### Spinal cord segmentation

Spinal cord segmentation relies on high contrast between WM and CSF. Using gradient-echo sequences, WM and CSF were previously measured to have largely different T1 (WM dorsal column: 853 ms, WM lateral column: 830 ms, CSF (estimated): 4000 ms)^34^ and also relative proton density values (WM: 0.7, CSF: 1)^35^ in the upper cervical cord at 3 T. Consequently, we observed an excellent WM/CSF contrast already in the first echo, also shown by Barry and Smith^17^. The radically different T2* values of these tissues (WM: 39.8 ms, CSF: ~ 2000 ms)^17^ further enhance the contrast at later echoes. However, while the WM/CSF contrast shows a steady increase with echo time, the CNR reaches a peak due to a steady decrease in SNR. Importantly, we show that combining successive echoes compensate for the SNR loss, yielding higher CSF/WM values than in individual echoes. This observation was especially pronounced in the lumbosacral enlargement. The quantitative results were in line with the qualitative assessment; combining a higher number of successive echoes steadily increased the CNR and also the ranking score for SC segmentability, with the combination of 5 echoes (all available echoes) yielding the highest values in both metrics. While combining successive echoes did not improve the SNR, raters still ranked combined echoes much higher in terms of SC segmentability, due to the improved clarity of the images. These findings underline that SC segmentation benefits from combining more echoes both in the lumbosacral enlargement and the conus medullaris. While not rated explicitly, combining more echoes also provides better visualization of the spinal roots and rootlets. We note that acquiring more than 5 echoes (i.e., echo times higher than 23 ms) is not likely to add further value to SC segmentation, as late echoes have low SNR and are susceptible to off-resonance artifacts. Most notably, signal dropout due to inhomogeneities in the static magnetic field often appears along the dorsal edge of the spinal cord, potentially compromising SC segmentation (Supplementary Fig. S3).

### Gray matter segmentation

Gray matter segmentation relies on good GM/WM contrast. GM and WM have different T1 (GM: 994 ms, WM dorsal column: 853 ms, WM lateral column: 830 ms) and relative proton density values (GM: 0.8, WM: 0.7), yielding a good contrast already in the first echo. However the contrast increases only slightly with increasing echo times due to similar T2* values at 3 T (GM: 41.3 ms, WM: 39.8 ms)^17,35^, which is not enough to compensate for the steady loss of SNR at later echoes (also demonstrated by Cohen-Adad et al.^36^). This overall leads to a steady decreased in CNR with increasing echo time (also shown in Barry and Smith^17^). Again, combining successive echoes yielded higher CNR than any of the individual echoes by compensating for the SNR loss, and also lead to better visibility of tissue edges as reported by the raters. Accordingly, echo combinations received much higher ranking scores for GM segmentability than individual echoes. While CNR between GM and WM were similar when combining 2, 3, 4, or 5 successive echoes, the highest ranking score was obtained when combining 3 successive echoes in the lumbosacral enlargement and 4 echoes in the conus medullaris. Combining even more echoes was perceived counter-productive by the raters, mostly due to the lower signal intensity and SNR within the GM.

### Recommendations

In light of the present results, when images are acquired with comparable echo times to this study, we recommend combining 5 successive echoes for segmenting the SC in the lumbosacral cord. For segmenting the GM, we recommend combining 3 echoes in the lumbosacral enlargement and 4 echoes in the conus medullaris. Importantly, echo times might vary depending on the sequence and vendor. As a rule of thumb, we recommend acquiring echoes until an echo time of around 15–18 ms. In our analyses, echoes acquired at echo times above this range (at 19 and 23 ms) did not improve GM segmentability further. Notably, these findings are in line with the results obtained in simulated cervical cord images, where CNR between GM/WM reached a plateau at echo times between 15 and 17 ms^36^. In any case, the shortest possible echo time, echo spacing, and repetition time should be selected to accommodate the maximum number of echoes and signal averages within the available imaging time. Beside the echo time, other key sequence parameters such as repetition time and flip angle also influence the contrasts. With the repetition time set to the minimum, the flip angle must be optimized for each sequence to provide a trade-off between WM/CSF and GM/WM contrast.

Instead of separate recommendations for SC and GM segmentations, the selection of a single optimal number of echoes is often necessary. First, while in principle SC and GM can be segmented in separate images, most studies utilizing multi-echo gradient-echo sequences aim to take advantage of the good WM/CSF and GM/WM contrasts by segmenting both the SC and GM in the same image. Second, several sequences such as MEDIC (Siemens) and MERGE (GE) automatically combine the echoes, providing the user with a single combined echo image. Therefore, the raters also assessed the combined echoes in terms of joint SC and GM segmentability. These findings were strikingly similar to those of the GM segmentability, with the combination of 3 and 4 echoes obtaining the highest ranking score in the lumbar enlargement and conus medullaris, respectively. Therefore, we recommend 3 echoes as the single optimal value, considering the drop in GM ranking score in the lumbosacral enlargement after 3 combined echoes This highlights an important difference between SC and GM segmentation: CNR between GM and WM contrast is several times lower (4–5 × in the lumbosacral enlargement, 2–3 × in the conus medullaris) than that between WM and CSF. This, coupled with the irregular shape of the GM makes GM segmentation a much more difficult task than SC segmentation. Therefore, the raters were rather willing to sacrifice on the WM/CSF contrast (while still maintaining a high value) in order to maximize GM/WM contrast.

While scan time scales linearly with the NSA, SNR and CNR grow more slowly. In the presence of pure thermal noise, SNR and CNR grow with the square root of NSA^31^. In this study, SNR and CNR, while still following an approximate square-root behavior, showed a less steep increase. For example, CNR GM/WM was found to be only 1.58 times (instead of twice) higher with 4 than with 1 signal average. This is probably due to the definition of noise in this study as the standard deviation of the signal within the tissues of interest, which contains both a stochastic (thermal noise) and a deterministic (intrinsic anatomical variations) component. Importantly, longer scan time also poses higher financial cost and burden on the patient and increases the risk for motion artifacts. The minimum NSA depends on the hardware, protocol, and participant. In our empirical analysis, six averages yielded good contrast for reliable GM and SC segmentation in all subjects, which corresponds to an acquisition time of 10:38 min when acquiring 3 echoes and 13:28 min when acquiring 5 echoes. To create signal averages, the acquired measurements can be averaged either in the scanner already outputting a single average image, or offline using image processing software. The second approach has two advantages: (i) the individual measurements can be individually inspected and removed if affected by artifacts, and (ii) motion correction can be applied to realign the measurements to a mid-point average, for example using SPM’s Serial Longitudinal Registration^37^. For higher SNR, it is possible to perform offline averaging on k-space data (if available), rather than on magnitude images; however, in practice, this may not always be feasible.

### Implications for clinical applications

Clinical applications aiming to quantify SC, GM and WM atrophy in the lower cord are likely to benefit from the present recommendations as they allow for accurate and precise segmentation of these tissue compartments in a cost-effective and time-efficient manner. Specifically, this study has demonstrated that successful segmentation of SC and GM can be achieved simultaneously within the same imaging volume, or independently, by using a different number of echoes. Future clinical applications could be tailored to address specific clinical questions within the shortest acquisition time by acquiring only the number of echoes required. Furthermore, although not required in this study, the option of co-registering individual repetitions post-acquisition minimizes the requirement of re-scans as a result of motion during acquisition. Image acquisition and analysis protocols offering reliable and precise tissue-specific segmentation are especially important as they can also reduce the sample size needed to achieve the desired effect size in clinical trials. The results from this study provide solid foundation toward standardized protocols in the lumbosacral cord.

### Generalizability

The presented recommendations on the optimal number of combined echoes are based on the Siemens 3D FLASH sequence acquired with a 3 T Siemens Prisma. 2D FLASH has some advantageous properties compared to its 3D counterpart such as less susceptibility to subject motion, more homogeneous B1 + profile, and no aliasing at the edges. Nevertheless, we expect to see similar qualitative behavior of SNR and CNR across echoes, as long as the echo times are identical, because the tissue parameters governing the contrasts and the signal equation are fundamentally the same in both sequences. However, the absolute values of SNR and CNR are expected to be higher in the 3D sequence due to its better SNR per time efficiency^20^. Given the potentially impactful differences in hardware and sequence implementation, multi-vendor studies are needed to investigate the generalizability of the presented recommendations to other vendors. Importantly, the available minimal echo time and echo spacing might be different in other sequences or vendors, as they depend on hardware and sequence parameters (e.g., the gradient field strength). Notably, driving the readout gradient in a unipolar mode (as opposed to bipolar mode used here) doubles the echo spacing and prolongs the echo times from the second echo upwards, with the benefit of avoiding the slight inter-echo shift in the k-space often occurring in bipolar mode. Therefore, the optimal number of echoes for GM and SC segmentability might be lower when using unipolar gradient mode.

Our empirical analyses focused on the lumbosacral cord, given the lack of consensus parameters in this region. In the cervical cord, segmentation on one hand benefits from the higher SNR due to the better coil coverage, but GM segmentation on the other hand is hampered by the smaller size of the GM and the corresponding larger contribution of partial volume effects. We expect the recommendations to hold in the cervical cord as well, as relaxation times governing the contrast have been measured to be similar at both levels^38^. Notably, the recently published consensus protocol for acquiring high-quality MRI data of the human cervical cord at 3 T also recommends the combination of three echoes (with the last echo acquired at 14 ms)^32^.

The optimal number of echo combinations also depends on the level of motion the individuals are prone to. The participants in this study presented negligible motion, hence no motion-related image artifacts, due to high compliance and our emphasis on subject preparation and positioning. In a less compliant population such as pediatric subjects or patients with particular neurological conditions, we anticipate higher levels of artifacts implying that the optimal number of echoes might decrease, as later echoes are more susceptible to motion artifacts due to longer read-out times^17^. B0 shimming performed with the scanner's linear and possibly higher order shim coils is essential to minimize intra-voxel dephasing due to susceptibility artifacts and to achieve proper fat saturation and clear slice excitation profiles. Advanced shimming techniques for the spinal cord, including z shimming^39,40^ and real-time shimming^41^, are currently in development, which are expected to render echoes above 15–18 ms beneficial for GM segmentation by reducing signal dropout. Application of navigator echoes (if available) likely improves the image quality of later echoes in the cervical cord by mitigating the ghosting associated with the time-varying magnetic fields around the spinal cord, most notably caused by the respiratory cycle^42,43^. Reduced ghosting can also improve the overall signal level inside the spinal cord in later echoes. This may make later echoes useful for analysis but comes at the cost of longer acquisition time. Furthermore, navigators may become unreliable in lower signal-to-noise regimes and in case of increased respiratory artifacts, potentially compromising their usage in the lumbosacral cord. A promising alternative to navigators is to base the correction on the signal from a respiratory bellow, which has been shown to substantially improve data quality in later echoes^44^. Consistent with the consensus acquisition protocol for cervical spinal cord gradient-echo images^32^, we do not recommend cardiac and respiratory gating, as they drastically increase the acquisition time for little expected gain.

### Considerations and limitations

SNR and CNR were not computed according to the classical definition^45^. Instead, the noise term for SNR was defined as the standard deviation of voxel intensities within the ROI (GM, WM, or CSF), and for CNR as the standard deviation of the difference of the voxel intensities between two ROIs^46–50^. Compared to the classical definition, these noise terms can be thought of as a measure of tissue uniformity, as they capture all sources of noise (thermal, physiological, bias field, etc.) as well as anatomical variations within the ROI. Therefore, we argue that they are more suitable to characterize segmentability, as manual segmentation benefits from high tissue uniformity without signal inhomogeneities, regardless of their source.

At typical resolutions (0.5 × 0.5 mm^2^ in-plane in our study), partial volume effects along the tissue interfaces lead to underestimation of SNR and CNR of the affected tissue. We tried to minimize partial volume effects by including voxels in the SC mask which lie fully inside the segmentation line and segmenting the GM in a conservative way. Remaining partial volume effects are especially relevant in the conus medullaris due to the small number of voxels; for example, the lower CNR between WM and CSF in the conus medullaris can be attributed in part to partial volume effects.

While several automatic SC and GM segmentation techniques have been shown to perform well in the cervical cord^47^, they have not yet been validated in the lumbosacral cord, leaving manual segmentation the gold standard segmentation technique in this region. To ensure an unbiased comparison of echoes, the same GM and SC segmentation were applied on all images of single echoes and echo combinations within the same subject. Note that for quantitative assessments, factors such as inter-rater, intra-rater, and scan-rescan variability have to be considered in addition to what has already been mentioned in this paper.

To conclude, multi-echo gradient-echo sequences are suitable for tissue-specific volumetric assessment in the lumbosacral cord. For reliable segmentation of the SC and GM within the same imaging volume, we suggest combining three successive echoes acquired using the shortest possible echo times. For SC or GM segmentation only, we recommend combining 5 or 3 successive echoes, respectively. Ideally, at least six individual signal averages should be acquired in order to achieve sufficiently high SNR and CNR for robust segmentation. The presented recommendations have great implications for clinical applications measuring SC, GM, and WM atrophy and represent a step toward standardized protocols in the lumbosacral cord.

## Supplementary Information

Supplementary Information.

## Data Availability

The datasets used and analyzed during the current study are available from the corresponding author on reasonable request.
