# Supplementary material for: Optimized multi-echo gradient-echo magnetic resonance imaging for gray and white matter segmentation in the lumbosacral cord at 3 T
Source: Sci Rep. 2022 Oct 3;12:16498. doi: 10.1038/s41598-022-20395-1 (PMC9530158; doi:10.1038/s41598-022-20395-1)
Supplement: Supplementary file 1 — Supplementary Information. [file 41598_2022_20395_MOESM1_ESM.docx]

Optimized multi-echo gradient-echo magnetic resonance imaging for gray and white matter segmentation in the lumbosacral cord at 3T

Silvan Büeler^1^, Marios C. Yiannakas^2^, Zdravko Damjanovski^1^, Patrick Freund^3,4,5^, Martina D. Liechti^1 +^, Gergely David^1,3 +*^

^+^ shared last authors

* corresponding author

1 Department of Neuro-Urology, Balgrist University Hospital, University of Zurich, Zurich, Switzerland

2 NMR Research Unit, Queen Square MS Centre, Department of Neuroinflammation, UCL Queen Square Institute of Neurology, University College London, United Kingdom

3 Spinal Cord Injury Center, Balgrist University Hospital, University of Zurich, Zurich, Switzerland

4 Department of Neurophysics, Max Planck Institute for Human Cognitive and Brain Sciences, Leipzig, Germany

5 Wellcome Trust Centre for Neuroimaging, UCL Queen Square Institute of Neurology, London, United Kingdom

**Supplementary information**

Table S1 lists mean ± standard deviation of $CNR_{WM/CSF}$, $CNR_{GM/WM}$, WM/CSF contrast, GM/WM contrast, ${SNR}_{GM}$, ${SNR}_{WM}$, $CNR_{WM/CSF}/\sqrt{t}$, and $CNR_{GM/WM}/\sqrt{t}$ across participants for individual and combined echoes. Table S2 lists mean ± standard deviation for $CNR_{WM/CSF}$, $CNR_{GM/WM}$, WM/CSF contrast, GM/WM contrast, ${SNR}_{GM}$, ${SNR}_{WM}$, $CNR_{WM/CSF}/\sqrt{t}$, and $CNR_{GM/WM}/\sqrt{t}$ across participants for different number of signal averages (1 to 8). Figure S3 shows examples of signal dropout at the dorsal edge of the spinal cord caused by magnetic field inhomogeneities (susceptibility artifacts).

Table S1 – Data for individual and combined echoes

Table S2 – Data for number of signal averages

Figure S3 – Examples of signal dropout

Supplementary Table S1: Mean values ± standard deviation across participants (n=10) for $CNR_{WM/CSF}$, $CNR_{GM/WM}$, WM/CSF contrast, GM/WM contrast, ${SNR}_{GM}$, and ${SNR}_{WM}$, $CNR_{WM/CSF}/\sqrt{t} (\%)$ and $CNR_{GM/WM}/\sqrt{t} (\%)$. Values are shown separately for individual echoes (1, 2, 3 ,4, and 5) and combined echoes (1-2, 1-3, 1-4 and 1-5) acquired with 8 signal averages. For each metric and subject, values were averaged across 3-3 slices in the LSE and the CM.

|  |  | **Echoes** | | | | | | | | |
| --- | --- | --- | --- | --- | --- | --- | --- | --- | --- | --- |
| **Metric** | **Region** | **1** | **2** | **3** | **4** | **5** | **1-2** | **1-3** | **1-4** | **1-5** |
| $CNR_{WM/CSF}$ | LSE | 4.65±1.52 | 5.03±1.55 | 5.04±1.58 | 4.85±1.56 | 4.44±1.69 | 5.68±1.85 | 6.15±2.06 | 6.40±2.04 | 6.59±2.20 |
|  | CM | 2.33±0.90 | 2.96±1.26 | 3.48±1.24 | 3.58±1.52 | 3.65±1.28 | 2.96±1.18 | 3.43±1.32 | 3.77±1.56 | 3.99±1.61 |
| $CNR_{GM/WM}$ | LSE | 1.63±0.17 | 1.51±0.18 | 1.38±0.24 | 1.19±0.23 | 1.06±0.23 | 1.77±0.18 | 1.80±0.21 | 1.78±0.22 | 1.76±0.22 |
|  | CM | 1.64±0.32 | 1.57±0.30 | 1.61±0.35 | 1.37±0.36 | 1.21±0.33 | 1.78±0.33 | 1.84±0.34 | 1.84±0.37 | 1.81±0.38 |
| ${Contrast}_{WM/CSF}$ (∙10^-1^) | LSE | 2.86±0.88 | 3.41±0.82 | 3.95±0.96 | 4.22±1.02 | 4.40±1.05 | 3.11±0.85 | 3.33±0.88 | 3.49±0.90 | 3.60±0.91 |
|  | CM | 1.54±0.60 | 2.06±0.78 | 2.64±0.83 | 3.13±0.89 | 3.69±0.64 | 1.78±0.69 | 2.00±0.75 | 2.18±0.81 | 2.36±0.80 |
| ${Contrast}_{GM/WM}$ (∙10^-1^) | LSE | 1.17±0.11 | 1.24±0.12 | 1.32±0.16 | 1.31±0.21 | 1.34±0.26 | 1.20±0.11 | 1.22±0.12 | 1.23±0.13 | 1.24±0.14 |
|  | CM | 1.15±0.25 | 1.26±0.25 | 1.41±0.32 | 1.45±0.31 | 1.43±0.23 | 1.19±0.24 | 1.24±0.25 | 1.27±0.26 | 1.28±0.25 |
| ${SNR}_{WM}$ | LSE | 19.3±1.9 | 16.8±1.7 | 13.9±1.6 | 12.4±1.5 | 10.8±1.4 | 20.6±1.8 | 20.4±2.0 | 20.2±2.1 | 19.9±2.1 |
|  | CM | 17.2±2.4 | 15.0±1.2 | 13.7±1.4 | 11.5±2.1 | 10.3±1.9 | 17.4±1.6 | 17.2±1.7 | 16.8±1.8 | 16.3±1.9 |
| ${SNR}_{GM}$ | LSE | 20.3±2.2 | 18.0±2.7 | 15.8±2.0 | 13.7±1.8 | 11.8±1.9 | 21.5±2.7 | 21.5±2.6 | 21.1±2.6 | 20.9±2.6 |
|  | CM | 26.3±3.3 | 22.5±2.9 | 21.0±3.0 | 17.6±2.4 | 15.7±2.8 | 28.5±4.0 | 28.9±3.4 | 28.4±3.6 | 28.2±3.9 |
| ${CNR}_{WM/CSF}/\sqrt{t} (\%)$ | LSE | 18.6±6.1 | 18.5±5.7 | 17.3±5.4 | 15.5±5.0 | 13.5±5.2 | 20.9±6.8 | 21.1±7.1 | 20.6±6.6 | 20.1±6.7 |
|  | CM | 9.3±3.6 | 10.9±4.7 | 12.0±4.3 | 11.5±4.9 | 11.14±3.91 | 10.9±4.4 | 11.8±4.5 | 12.1±5.0 | 12.2±4.9 |
| ${CNR}_{GM/WM}/\sqrt{t} (\%)$ | LSE | 6.5±0.7 | 5.6±0.7 | 4.7±0.8 | 3.8±0.7 | 3.2±0.7 | 6.5±0.7 | 6.2±0.7 | 5.7±0.7 | 5.4±0.7 |
|  | CM | 6.6±1.3 | 5.8±1.1 | 5.5±1.2 | 4.4±1.2 | 3.7±1.0 | 6.5±1.2 | 6.3±1.2 | 5.9±1.2 | 5.5±1.2 |

Abbreviations: CNR = contrast-to-noise ratio, SNR = signal-to-noise ratio, CSF = cerebrospinal fluid, WM = white matter, GM = gray matter, LSE = lumbosacral enlargement, CM: conus medullaris, t = acquisition time (in seconds).

Supplementary Table S2: Mean values ± standard deviation across participants (n=10) for $CNR_{WM/CSF}$, $CNR_{GM/WM}$, WM/CSF contrast, GM/WM contrast, ${SNR}_{GM}$, ${SNR}_{WM}$, $CNR_{WM/CSF}/\sqrt{t} (\%)$ and $CNR_{GM/WM}/\sqrt{t} (\%)$. Values are shown separately for different number of signal averages (1 to 8), in each case taking the combination of 3 echoes. For each metric and subject, values were averaged across 3-3 slices in the LSE and the CM.

|  |  | **Number of signal averages** | | | | | | | |
| --- | --- | --- | --- | --- | --- | --- | --- | --- | --- |
| **Metric** | **Region** | **1** | **2** | **3** | **4** | **5** | **6** | **7** | **8** |
| $CNR_{WM/CSF}$ | LSE | 2.94±1.24 | 3.96±1.68 | 4.43±1.77 | 4.82±1.96 | 5.23±1.95 | 5.59±1.98 | 5.53±1.98 | 6.07±2.10 |
|  | CM | 1.83±0.69 | 2.27±1.00 | 2.67±1.22 | 2.73±1.30 | 2.85±1.26 | 3.07±1.30 | 3.15±1.29 | 3.32±1.43 |
| $CNR_{GM/WM}$ | LSE | 0.90±0.26 | 1.15±0.27 | 1.33±0.32 | 1.42±0.35 | 1.53±0.36 | 1.60±0.34 | 1.59±0.35 | 1.70±0.35 |
|  | CM | 0.95±0.33 | 1.19±0.41 | 1.35±0.43 | 1.46±0.43 | 1.55±0.46 | 1.63±0.52 | 1.63±0.55 | 1.71±0.58 |
| ${Contrast}_{WM/CSF}$ (∙10^-1^) | LSE | 3.03±0.88 | 3.03±0.86 | 3.02±0.75 | 3.04±0.71 | 3.07±0.68 | 3.11±0.70 | 3.11±0.71 | 3.15±0.72 |
|  | CM | 2.03±0.85 | 1.93±0.75 | 2.01±0.73 | 1.93±0.82 | 1.91±0.75 | 1.92±0.75 | 1.95±0.74 | 1.95±0.78 |
| ${Contrast}_{GM/WM}$ (∙10^-1^) | LSE | 1.11±0.24 | 1.14±0.24 | 1.17±0.27 | 1.17±0.28 | 1.19±0.26 | 1.19±0.23 | 1.19±0.21 | 1.20±0.21 |
|  | CM | 1.10±0.33 | 1.11±0.34 | 1.13±0.32 | 1.16±0.33 | 1.17±0.33 | 1.16±0.36 | 1.16±0.37 | 1.16±0.38 |
| ${SNR}_{WM}$ | LSE | 10.9±1.4 | 13.9±1.6 | 15.7±1.9 | 16.6±1.9 | 18.0±1.8 | 18.9±1.9 | 18.7±2.1 | 19.8±1.9 |
|  | CM | 10.8±2.0 | 12.9±1.9 | 14.2±1.7 | 14.9±1.8 | 15.6±2.1 | 16.3±2.3 | 16.4±2.4 | 16.8±2.3 |
| ${SNR}_{GM}$ | LSE | 11.7±1.3 | 14.8±1.1 | 16.8±1.9 | 18.0±2.2 | 19.0±2.0 | 19.6±2.2 | 19.5±2.4 | 20.8±2.4 |
|  | CM | 14.3±3.3 | 19.1±3.2 | 21.7±3.6 | 23.9±4.0 | 25.3±4.3 | 26.1±4.8 | 26.2±5.0 | 27.8±5.4 |
| ${CNR}_{WM/CSF}/\sqrt{t} (\%)$ | LSE | 28.3±11.9 | 27.1±11.5 | 24.7±9.9 | 23.4±9.5 | 22.7±8.4 | 22.1±7.9 | 20.3±7.2 | 20.8±7.2 |
|  | CM | 17.7±6.7 | 15.5±6.9 | 14.9±6.8 | 13.2±6.3 | 12.3±5.4 | 12.2±5.2 | 11.6±4.7 | 11.4±4.9 |
| ${CNR}_{GM/WM}/\sqrt{t} (\%)$ | LSE | 8.7±2.5 | 7.9±1.8 | 7.4±1.7 | 6.9±1.7 | 6.7±1.6 | 6.4±1.4 | 5.9±1.3 | 5.8±1.2 |
|  | CM | 9.2±3.1 | 8.1±2.8 | 7.5±2.4 | 7.1±2.1 | 6.7±2.0 | 6.4±2.1 | 6.0±2.03 | 5.9±2.0 |

Abbreviations: CNR = contrast-to-noise ratio, SNR = signal-to-noise ratio, CSF = cerebrospinal fluid, WM = white matter, GM = gray matter, LSE = lumbosacral enlargement, CM: conus medullaris, t = acquisition time (in seconds).


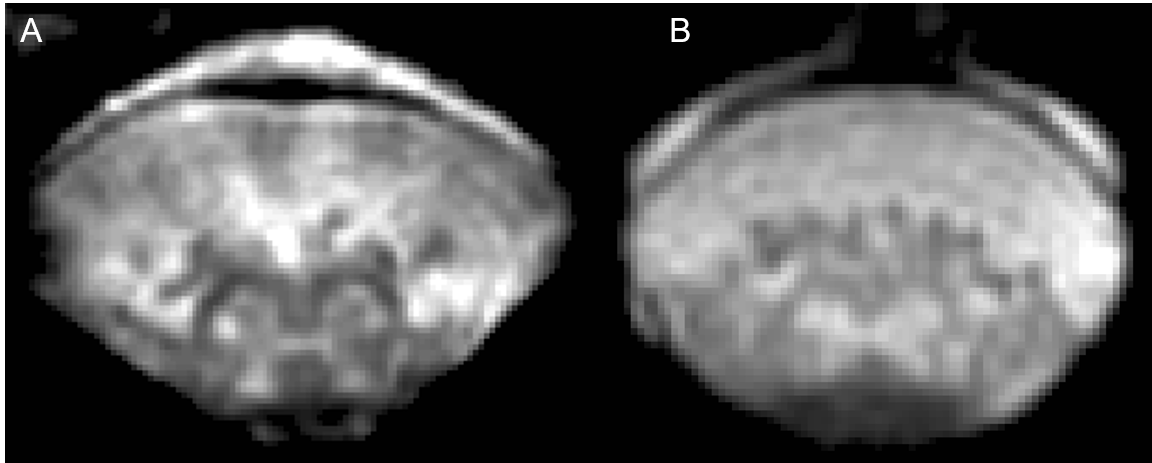


Supplementary Figure S3: Signal dropout at the dorsal edge of the spinal cord caused by magnetic field inhomogeneities (susceptibility artifacts). A) Representative slice in the lumbosacral enlargement. B) Representative slice in the conus medullaris.
